# Age- and Light-Dependent Development of Localised Retinal Atrophy in CCL2^−/−^CX3CR1^GFP/GFP^ Mice

**DOI:** 10.1371/journal.pone.0061381

**Published:** 2013-04-18

**Authors:** Mei Chen, Jose R. Hombrebueno, Chang Luo, Rosana Penalva, Jiawu Zhao, Liza Colhoun, Sudha Pirya Soundara Pandi, John V. Forrester, Heping Xu

**Affiliations:** 1 Centre for Vision and Vascular Science, School of Medicine, Dentistry and Biomedical Sciences, Queen’s University Belfast, Belfast, Northern Ireland, United Kingdom; 2 Ocular Immunology Group, Section of Immunology and Infection, School of Medicine and Dentistry, University of Aberdeen, Aberdeen, Scotland, United Kingdom; University of Cologne, Germany

## Abstract

Previous studies have shown that CCL2/CX3CR1 deficient mice on C57BL/6N background (with rd8 mutation) have an early onset (6 weeks) of spontaneous retinal degeneration. In this study, we generated CCL2^−/−^CX3CR1^GFP/GFP^ mice on the C57BL/6J background. Retinal degeneration was not detected in CCL2^−/−^CX3CR1^GFP/GFP^ mice younger than 6 months. Patches of whitish/yellowish fundus lesions were observed in 17∼60% of 12-month, and 30∼100% of 18-month CCL2^−/−^CX3CR1^GFP/GFP^ mice. Fluorescein angiography revealed no choroidal neovascularisation in these mice. Patches of retinal pigment epithelium (RPE) and photoreceptor damage were detected in 30% and 50% of 12- and 18-month CCL2^−/−^CX3CR1^GFP/GFP^ mice respectively, but not in wild-type mice. All CCL2^−/−^CX3CR1^GFP/GFP^ mice exposed to extra-light (∼800lux, 6 h/day, 6 months) developed patches of retinal atrophy, and only 20–25% of WT mice which underwent the same light treatment developed atrophic lesions. In addition, synaptophysin expression was detected in the outer nucler layer (ONL) of area related to photoreceptor loss in CCL2^−/−^CX3CR1^GFP/GFP^ mice. Markedly increased rhodopsin but reduced cone arrestin expression was observed in retinal outer layers in aged CCL2^−/−^CX3CR1^GFP/GFP^ mice. GABA expression was reduced in the inner retina of aged CCL2^−/−^CX3CR1^GFP/GFP^ mice. Significantly increased Müller glial and microglial activation was observed in CCL2^−/−^CX3CR1^GFP/GFP^ mice compared to age-matched WT mice. Macrophages from CCL2^−/−^CX3CR1^GFP/GFP^ mice were less phagocytic, but expressed higher levels of iNOS, IL-1β, IL-12 and TNF-α under hypoxia conditions. Our results suggest that the deletions of CCL2 and CX3CR1 predispose mice to age- and light-mediated retinal damage. The CCL2/CX3CR1 deficient mouse may thus serve as a model for age-related atrophic degeneration of the RPE, including the dry type of macular degeneration, geographic atrophy.

## Introduction

Age-related macular degeneration (AMD) is a disease, in which the neuronal retina of the macula gradually degenerates with age resulting in the loss of central vision [1]. Clinically, the disease presents with the accumulation of drusen, yellowish deposits in the macula between the retinal pigment epithelium (RPE) and Bruch’s membrane [1], or reticular pseudo-drusen at the subretinal space [2], [3]. The disease may progress to two advanced forms, geographic atrophy (dry AMD) where gradual vision loss occurs due to RPE and photoreceptor cell death, and neovascular AMD (wet AMD) where visual loss is sudden due to haemorrhage or fluid leakage from a subretinal neovascular membrane. The pathogenesis of AMD is not fully understood.

Compelling evidence suggests that inflammation may play a causal role in AMD [4]–[6]. Genetic studies have shown that the risk of AMD is related to polymorphisms of a number of immune related genes, including complement factor H (*CFH)* [7]–[10], complement component 2 and factor B *(C2/CFB)* [11], complement component 3 *(C3)* [12]–[14], Toll-like receptor *(TLRs)* [15], and fractalkine receptor *(CX3CR1)* genes[16]–[19], although a recent study by Zerbib et al did not confirm the association between CX3CR1 polymorphism (T280M) and neovascular AMD in a French population [20]. Drusen, the hall-mark of early AMD, contains many components that can stimulate an inflammatory response in the RPE/Brusch’s membrane layer, with recruitment of macrophages [21], [22]. A recent study has shown that anti-inflammatory therapy in combination with anti-VEGF treatment may provide additional benefits (as compared to anti-VEGF alone) to wet-AMD [23] However, the underlying mechanism related to inflammation in AMD remains ill-defined.

We have shown previously that a para-inflammatory response, characterized by microglial activation and subretinal migration, and complement activation, is a part of normal retinal homeostasis during aging [24]–[26]. Under chronic stress conditions, retinal para-inflammation may also involve the recruitment of bone marrow-derived myeloid cells induced by CCL2-CCR2 ligation [27]. In the absence of CCL2 or CCR2, age-related retinal para-inflammation is dysregulated, and is related to the development of dry AMD-like lesions in these mice [28], [29]. Furthermore, mice deficient in CX3CR1 age-dependently develop AMD-like lesions [16], and mice deficient in both CCL2 and CX3CR1 have an early onset of retinal degeneration [30], although recent studies suggest that pathologies in the CCL2/CX3CR1 double knockout (DKO) mice is related to the Crb1 rd8 mutation [31], [32]. The above evidence suggests that the malfunction of the chemokine ligand-receptor pairs CCL2-CCR2, or CX3CL1-CX3CR1 may be related to the dysregulated retinal para-inflammation and retinal lesion development under aging conditions. In contrast to the above observations, other studies failed to detect retinal lesions in these mice. Luhmann et al observed increased subretinal macrophage accumulation but no retinal degeneration in aged (up to 25 months) CCL2 KO mice [33]. The group also reported no retinal degeneration in 12–14 months old CCL2 KO, CX3CR1 KO and CCL2/CX3CR1 DKO mice housed under dim conditions (33 lux light intensity) [34]. Vessey et al, however, observed inner retinal (amacrine cell) dysfunction in 9-month old CCL2/CX3CR1^gfp/gfp^ mice [35]. The role of the CCL2-CCR2 and CX3CL1-CX3CR1 chemokine ligand-receptor pairs in the age-related retinal para-inflammatory response and their contribution to age-related AMD-like retinal lesion development warrant further investigation.

In this study, we generated the double knockout (DKO) CCL2^−/−^CX3CR1^GFP/GFP^ reporter mice on the C57/BL6/J background which does not express the rd8 mutation. The mice are deficient in both CCL2 and CX3CR1, and express GFP in myeloid-derived cells, which readily allows myeloid cell detection. We show that the CCL2^−/−^CX3CR1^GFP/GFP^ mice age-dependently develop localised RPE and photoreceptor atrophy akin to the human geographic atrophy (GA) dry type of AMD. More importantly, we found that GA-like lesions in these mice can be further enhanced by environmental risk factors such as light. Retinal lesion development is related to enhanced microglial activation and altered myeloid cell function in these mice.

## Materials and Methods

### Generation of CCL2^−/−^CX3CR1^GFP/GFP^ Mice

CCL2^−/−^ mice (B6.129S4-*Ccl2^tm1Rol^*/J, Jackson Laboratory, Bar Harbor, USA) and CX3CR1^GFP/GFP^ mice [36] were used as founder generations (F0). The offspring mice (F1) were intercrossed to obtain CCL2^−/−^CX3CR1^GFP/GFP^ mice. Mice genotypes were confirmed by PCR. DNA sequencing confirmed that all mice (WT, F0 founders and CCL2^−/−^CX3CR1^GFP/GFP^ mice) do not have Crb1 gene rd8 mutation.

CCL2^−/−^CX3CR1^GFP/GFP^ mice were maintained in standard animal housing rooms with 12 h light-dark cycle. In some experiment, 4 months old mice were exposed to ∼800 lux during their 12 h-light cycle, 6 h/day for 6 months. All in vivo procedures were under the regulation of UK Home Office Animals (Scientific Research) Act 1986, and approved by the Ethical Review Committee of Queen’s University Belfast (Project Licence Number: PPL 2664). The study was conducted in compliance with the Association for Research in Vision & Ophthalmology Statement for the Use of Animals in Ophthalmology and Vision Research.

### Clinical Investigations

Fundus imaging: A topic endoscopic fundus imaging (TEFI) system described previously [37], [38] was used to obtain fundus images at different times (1.5, 3, 6, 9, 12, and 18 month). Images were captured using the Nikon D90 camera and saved in TIFF format. Adobe Photoshop (Version 6.0) software was used to adjust image brightness and contrast.

Indocyanine green (ICG) angiography: Mice were anesthetized with ketamine hydrochloride (60 mg/kg, ip, Fort George Animal Centre, Southampton, United Kingdom), and xylazine (5 mg/kg, ip, Pharmacia & Veterinary Products, Kiel, Germany). Pupils were dilated using 1% tropicamide and 2.5% phenylephrine (Chauvin, Essex, UK). 100 µl of 2.5% of ICG (American Biochemical & Pharmaceuticals Ltd, Surrey, UK) was injected through the tail vein, and angiographic images were captured using the HRA2 system (Heidelberg Engineering Ltd, Hemel Hempstead, UK).

### Induction of Choroidal Neovascularisation

Mice were anesthetised and pupils were dilated as above described. Three 532 diode laser spots (100 µm spot size, 100 msec, 200 mW) were applied to each fundus to induce choroidal neovascularisation (CNV). The rupture of Bruch’s membrane was confirmed by the production of a vaporization bubble at the time of laser treatment. Ten days later, mice were sacrificed and eyes were harvested for RPE/choroidal flatmount investigation (see below).

### Retinal Pigment Epithelial cell Isolation and Culture

RPE cells were isolated from 3-month old WT and DKO mice using a protocol described previously [39], [40]. In brief, retinas were removed from mouse eye-cups. The eyecups were then immersed in 0.5% trypsin-EDTA (INC Flow, Irvine, CA) at 37°C for 45 minutes. RPE cells were released from eyecups by gentle aspiration. The detached RPE cells were then cultured in DMEM supplemented with 10% fetal calf serum (Sigma, Cambridge, UK) for 6 days and passaged when reaching confluence. 3^rd^ passage RPE cells were used to detect VEGF-165 mRNA expression.

### Retinal or RPE/Choroidal Flatmount Preparation

Mouse eyes were fixed with 2% paraformaldehyde (Agar Scientific Ltd. Cambridge, UK) for 2 h. Retina and RPE/choroid were then dissected and processed for wholemount staining as previously described [41], [42]. In brief, retinal tissues were permeabilized with 0.3% triton X-100 for 2∼4 h and RPE/choroidal tissues for 1 h at room temperature. Retinal tissues were then incubated with biotin-conjugated isolectin B4 (1∶100 in 0.1% triton X-100/PBS, Vector Lab, USA) overnight, followed by R-PE conjugated straptavidine (1∶100, Invitrogen, Paisley, UK). RPE/choroidal tissues from normal aging eyes were incubated with Rhodamine-conjugated phalloidin (1∶100, Invitrogen Molecular Probes, Paisley, UK) at 4°C for 3 h. RPE/choroidal tissues from CNV-induced eyes were incubated with biotinylated isolectin B4 (1∶100, Vector Laboratories Ltd, Peterborough, UK) and rabbit anti-mouse collagen IV (1∶100, ABD Serotec Ltd. Oxford, UK) in 0.1% Triton X-100 at 4°C, overnight, followed by FITC-streptavidin (1∶200, Vector Laboratories Ltd) and goat anti-rabbit Alex Fluor 594 (1∶200, Invitrogen, Paisley, UK) for 2 h at room temperature. After thorough washes, samples were flat-mounted on glass slides and examined by confocal microscopy (C1 Nikon Confocal Microscope, Eclipse TE200-U, Nikon UK Ltd, Surry, UK). Confocal images were analysed using the NIS Element (Nikon UK Ltd, Surry, UK) imaging analysis software.

### Immunohistochemistry

Mouse eyes were fixed with 2% paraformaldehyde for 2 h. Eyes were then transferred to a 30% sucrose solution in PBS for 24 h at 4°C and embedded in Optimum Cutting Temperature (OCT) media (Sakura Finetek, Torrance, USA). Fourteen micrometer thick cryosections were blocked with 10% fetal calf serum (FCS) in 1% Triton X-100/PBS for 1 h. Sections were then incubated with rabbit polyclonal anti-cone arrestin (AB15282, Chemicon; Temecula, CA dilution 1∶10000), mouse monoclonal anti-rhodopsin (clone 4D2, Chemicon; dilution 1∶500), rabbit polyclonal anti-human synaptophysin (N1566, Dako UK Ltd, Cambridge, UK; dilution 1∶200), rabbit polyclonal anti-glial fibrillary acidic protein (GFAP, AB7260, Abcam, Cambridge, UK; dilution 1∶500), guinea pig polyclonal anti-γ-aminobutyric acid (GABA, AB175, Chemicon; dilution 1∶500) or goat polyclonal anti-glycine transporter 1 (GlyT1, AB1770, Chemicon; dilution 1∶5000) overnight at 4°C. After washing, sections were incubated for 2 h with Alexa Fluor 594 donkey anti-rabbit IgG (1∶200, Invitrogen) or Alexa Fluor 488 donkey anti-mouse IgG (1∶200, Invitrogen). Samples were cover-slipped with Vectashield mounting medium with DAPI (Vector Laboratories) and examined by confocal microscopy (C1 Nikon Confocal Microscope, Eclipse TE200-U).

### Histology

Mouse eyes were fixed in 2.5% glutaraldehyde for at least 3 days. Eye-cups were dissected into small pieces, and post-fixed in 1% osmium tetroxide in sodium cacodylate buffer for 1 h. Samples were then dehydrated through ascending ethanol washes (50% to 100%) and infiltrated with 100% agar low viscosity resin (LVR, Agar) overnight. Samples were embedded in LVR and incubated at 60°C for 48 h. Semithin sections (1 µm) were cut using the Leica Ultracut UCT microtome (Leica Microsyetems Ltd. Milton Keynes, UK), and stained with 1% toluidine blue (Sigma-Aldrich), and examined by light microscopy (Nikon Eclipse E400).

Ultrathin sections (80 nm) were prepared for transmission electron microscopy. Sections were loaded onto 200 mesh copper grids and stained with uranyl acetate saturated solution and 2.5% lead citrate. Samples were visualized using a FEI Philips CM 100 transmission electron microscope (FEI, Eindhoven, the Netherlands) operating at 100 kV.

### Culture of Bone Marrow-derived Macrophages

Bone marrow-derived macrophages (BMDMs) were cultured as previously described [28]. In brief, bone marrow cells were collected from mouse tibias and femurs and red blood cells were removed with lysis buffer (0.75% NH4Cl, 0.2% Tris–HCl, PH 7.2). Cells were then cultured in Dulbecco’s modification of Eagle’s medium (DMEM; Gibco BRL, Paisley, UK) supplemented with 15% fetal calf serum (Sigma-Aldrich) and 15% L929 conditioned medium at 37°C in 5% CO2 incubator for 5 days. BMDMs (M0) were polarized into M1 (by 50 ng/ml LPS, Sigma-Aldrich) or M2 (by 20 ng/ml IL4, R&D systems, UK) macrophages. In some experiments, BMDMs were cultured in 1% oxygen chamber for 8 h prior total RNA extraction.

### Phagocytosis Assay

BMDMs were seeded into a 96-well plate at the density of 1×10^5^/200 µl/well). The phagocytosis assay was performed using the pHrodo™ E Coli Bioparticles Kit (Invitrogen) according to the manufacturer’s instructions. Fluorescence intensity was measured at different time points using Tecan Microplate reader (Tecan Group Ltd. Mannedorf, Switzerland) with 550 nm excitation wavelength and a 600 nm emission filter. Bioparticle conjugates alone acted as a background control. Net phagocytosis was calculated by subtracting the average fluorescence intensity of the negative control wells (particles without cells) from the experimental wells.

### Flow Cytometry

Blood samples were collected from 3- and 18-month old CCL2^−/−^CX3CR1^GFP/GFP^ mice and WT C57/BL6J mice. 50 µl volumes of blood were incubated with antibody cocktails containing rat anti-mouse MCH class II (I-A/I-E, M5/114.15.2), CD45 (30-F11), CD4 (RM4-5) and CD8a (53-6.7), B220 (RA3-6B2), CD11b (GL1), Gr-1 (RB6-8C5) (1∶100; all from BD Biosciences) and PE-anti mouse F4/80 (1∶100, CI:A3-1, AbD Serotec). Red blood cells were then removed with lysis buffer. Data were collected using the BD FACSCanto II (BD Biosciences) and analysed using FlowJo software (Tree Star Inc). CD45^+^ cells were gated from the live cell population (SSC versus FSC). Leukocyte subsets (CD4, CD8, B220, CD11b and Gr1) were then gated from CD45^+^ cells.

### Real-time RT-PCR

Total RNA was extracted from retinal tissue by RNeasy Mini kit (Qiagen Ltd, Crawley, UK) and from cultured cells by Trireagent (Sigma-Aldrich, Poole, UK) according to the manufacturer’s instructions. The quantity and quality of RNA were determined using a NanoDrop ND-1000 spectrophotometer (NanoDrop Technologies, Wilmington, DE). The same amount of total RNA was used for reverse transcription using SuperScrip™ II Reverse Transcriptase kit and random primers (Invitrogen).

Real-time RT-PCR was performed using SYBR Green Master (Roche Diagnostics GmbH, Mannheim, Germany) in LightCycler® 480 system (Roche Diagnostics GmbH). The primer sequences are listed in table 1. Beta-actin and 18s were used as retinae and BMDMs housekeeping controls respectively.

**Table 1:** Primer sequences for real time RT-PCR.

Genes	Forward	Reverse
*C1qb*	ATAAAGGGGGAGAAAGGGCT	CGTTGCGTGGCTCATAGTT
*C3*	AGCAGGTCATCAAGTCAGGC	GATGTAGCTGGTGTTGGGCT
*C4*	ACCCCCTAAATAACCTGG	CCTCATGTATCCTTTTTGGA
*CFH*	CGTGAATGTGGTGCAGATGGG	AGAATTTCCACACATCGTGGCT
*CFB*	CTCCTCTGGAGGTGTGAGCG	GGTCGTGGGCAGCGTATTG
*C1INH*	TTGAGTGCCAAGTGGAAGATAAC	GTGCTTTGGGAACACGGGTAC
*CD59a*	TGTCTAGAGCAGGATCTAGC	ATCCGTCACTTTTGTTACAC
*IL-10*	TGCAGGACTTTAAGGGTTACTTGG	GGCCTTGTAGACACCTTGGTC
*IL-1b*	TCCTTGTGCAAGTGTCTGAAGC	ATGAGTGATACTGCCTGCCTGA
*IL-6*	TCTGCAAGAGACTTCCATCCAGT	TCTGCAAGTGCATCATCGTTGT
*IL12P40*	GACATCATCAAACCAGACCCGCC	GCCTTTGCATTGGACTTCGGT
*TNF-a*	GCCTCTTCTCATTCCTGCTT	CTCCTCCACTTGGTGGTTTG
*iNOS*	GGCAAACCCAAGGTCTACGTT	TCGCTCAAGTTCAGCTTGGT
*Arg1*	TTATCGGAGCGCCTTTCTCAA	TGGTCTCTCACGTCATACTCTGT
*VEGFa*	CCCACGTCAGAGAGCAACAT	TTTCTTGCGCTTTCGTTTTT
*CCL2*	AGGTCCCTGTCATGCTTCTG	TCTGGACCCATTCCTTCTTG
*CCL5*	ACTCCCTGCTGCTTTGCCTAC	GAGGTTCCTTCGAGTGACA
*CXCL2*	AAGTTTGCCTTGACCCTGAA	AGGCACATCAGGTACGATCC
*CXCL10*	GGATGGCTGTCCTAGCTCTG	ATAACCCCTTGGGAAGATGG
*CXCL12*	AAACCAGTCAGCCTGAGCTACC	GGCTCTGGCGATGTGGC
*CX3CL1*	ACGAAATGCGAAATCATGTGC	CTGTGTCGTCTCCAGGACAA
*NLRP3*	ATTACCCGCCCGAGAAAGG	TCGCAGCAAAGATCCACACAG
*18s*	AGGGGAGAGCGGGTAAGAGA	GGACAGGACTAGGCGGAACA
*β-actin*	CCTTCCTTCTTGGGTATG	TGTAAAACGCAGCTCAGTAA

## Results

### CCL2^−/−^CX3CR1^GFP/GFP^ mice Develop Dry-AMD Like Changes with Age

**Figure 1:**
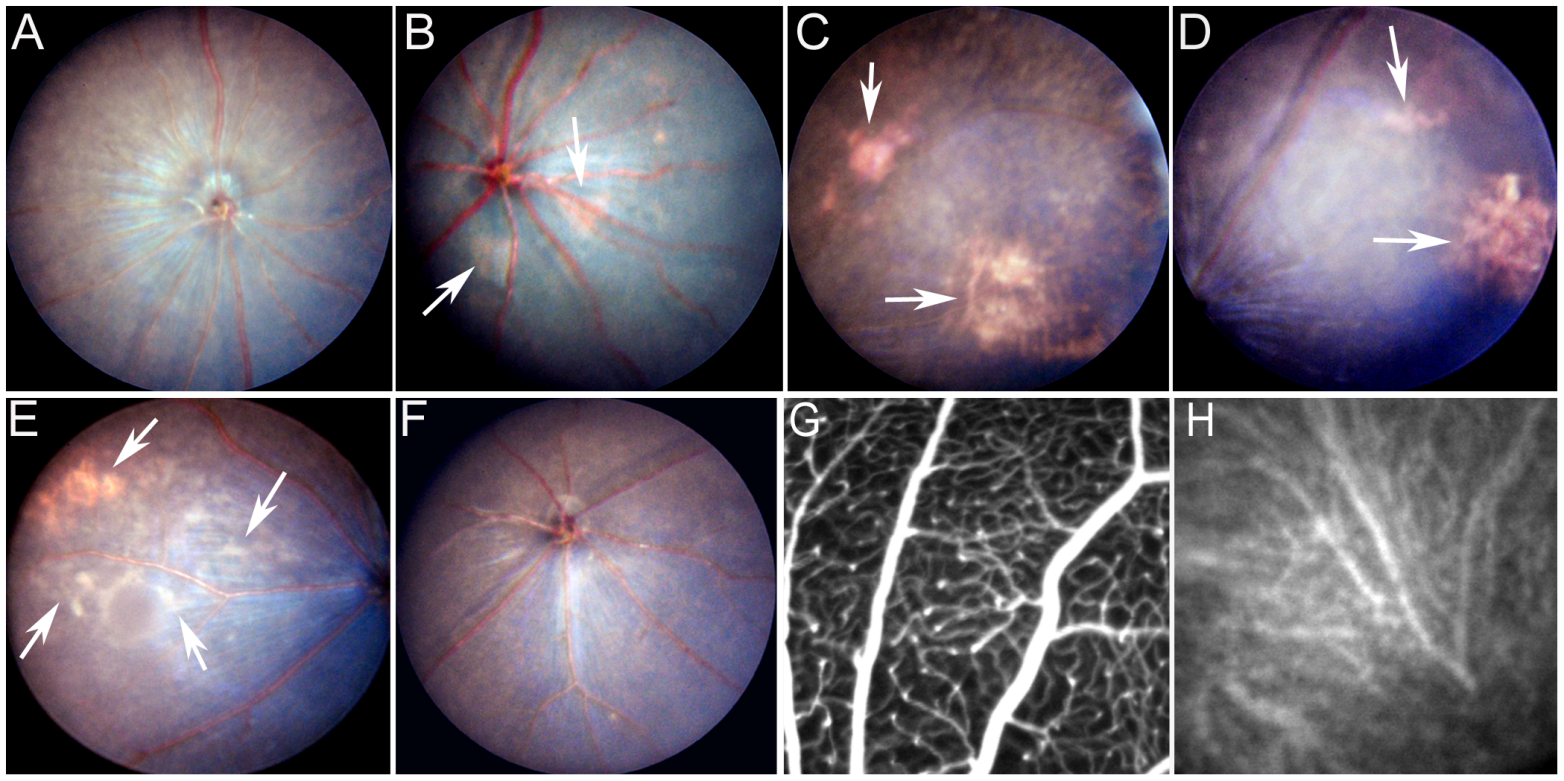
Retinal lesion in CCL2^−/−^CX3CR1^GFP/GFP^ mice. (A–F) Fundus image from 6-month (A), 12-month (B, C), 18-month (D), 10-month+light (E) CCL2^−/−^CX3CR1^GFP/GFP^ mice, and 18-month WT mice (F). Arrows indicate retinal lesions. (G, H) Indocyanine green angiography showing retinal (G) and choroidal (H) circulation of an 18-month old CCL2^−/−^CX3CR1^GFP/GFP^ mouse. CNV was not observed in CCL2^−/−^CX3CR1^GFP/GFP^ mice.

Under normal housing conditions, no fundus abnormalities were observed in 3∼6 months old CCL2^−/−^CX3CR1^GFP/GFP^ mice (Figure 1A). Patches of yellowish/whitish lesions were observed in 17–60% of 12-month old (Figure 1B, 1C), and 30–100% of 18-month old (Figure 1D) CCL2^−/−^CX3CR1^GFP/GFP^ mice in three studies (Table 2). The majority of lesions were detected in the temporal area (both upper and lower) between optic disc and equatorial region of the retina, and lesion size varies from 0.5∼5 optic disc diameter (Figure 1B–1E). Lesions were rarely detected in the peripheral retinal areas. Light is considered one of the main risk factors for AMD [43], [44]. The average light intensity inside the housing room was 200 lux. However, cages at different locations received different levels of light exposure. The average light intensity inside the cages located at the bottom layer of the rack was 46 lux (12–125 lux), whereas the cages located at the top layer had 180 lux (58–380 lux) light exposure. The levels of light intensity inside the cage positively correlated to the incidence of fundus lesion (Pearson, r = 0.993, P = 0.006, Table 2). None of the WT mice developed retinal atrophic lesions under normal housing conditions (Table 2).

**Table 2:** Retinal lesion in different experiments under normal housing conditions.

Exp	Mice (No. & strain)	Average light intensity (lux)*	No. of mice with retinal lesion at 12 months (%)	No. of mice with retinal lesion at 18 months (%)
**1**	WT: n = 12	46 (n = 6)	0 (0%)	0 (0%)
		98 (n = 6)	0 (0%)	0 (0%)
	DKO: n = 12	46 (n = 6)	1 (17%)	2 (30%)
		98 (n = 6)	3 (50%)	3 (50%)
**2**	WT: n = 7	120	0 (0%)	0 (0%)
	DKO: n = 6	120	2 (30%)	4 (67%)
**3**	WT: n = 10	180	0 (0%)	0 (0%)
	DKO: n = 10	180	6 (60%)	10 (100%)

* , Light intensity was the average of measurements taken from 5 different areas inside the cage: front, middle, rear, under the food, under the water box.

The effect of light on retinal lesion development was further confirmed by extra light exposure. When mice were exposed to ∼800 lux light 6 h/day for 6∼7 months, 83–100% of CCL2^−/−^CX3CR1^GFP/GFP^ mice developed patches of yellowish and whitish lesions (Table 3, Figure 1E). Interestingly 20–25% of WT mice also developed retinal lesions after 800 lux chronic light treatment (Table 3).

**Table 3:** Retinal lesion in mice that were exposed to 800lux light.

Exp	Mice (No. & strain)	Light treatment start age	No. of mice with retinal lesion at 6 months (%)	No. of mice with retinal lesion at 10 months (%)
**1**	WT: n = 10	4-month	0 (0%)	2 (20%)
	DKO: n = 12	4-month	3 (25%)	10 (83%)
**2**	WT: n = 8	3-month	0 (0%)	2 (25%)
	DKO: n = 8	3-month	4(50%)	8 (100%)

**Figure 2:**
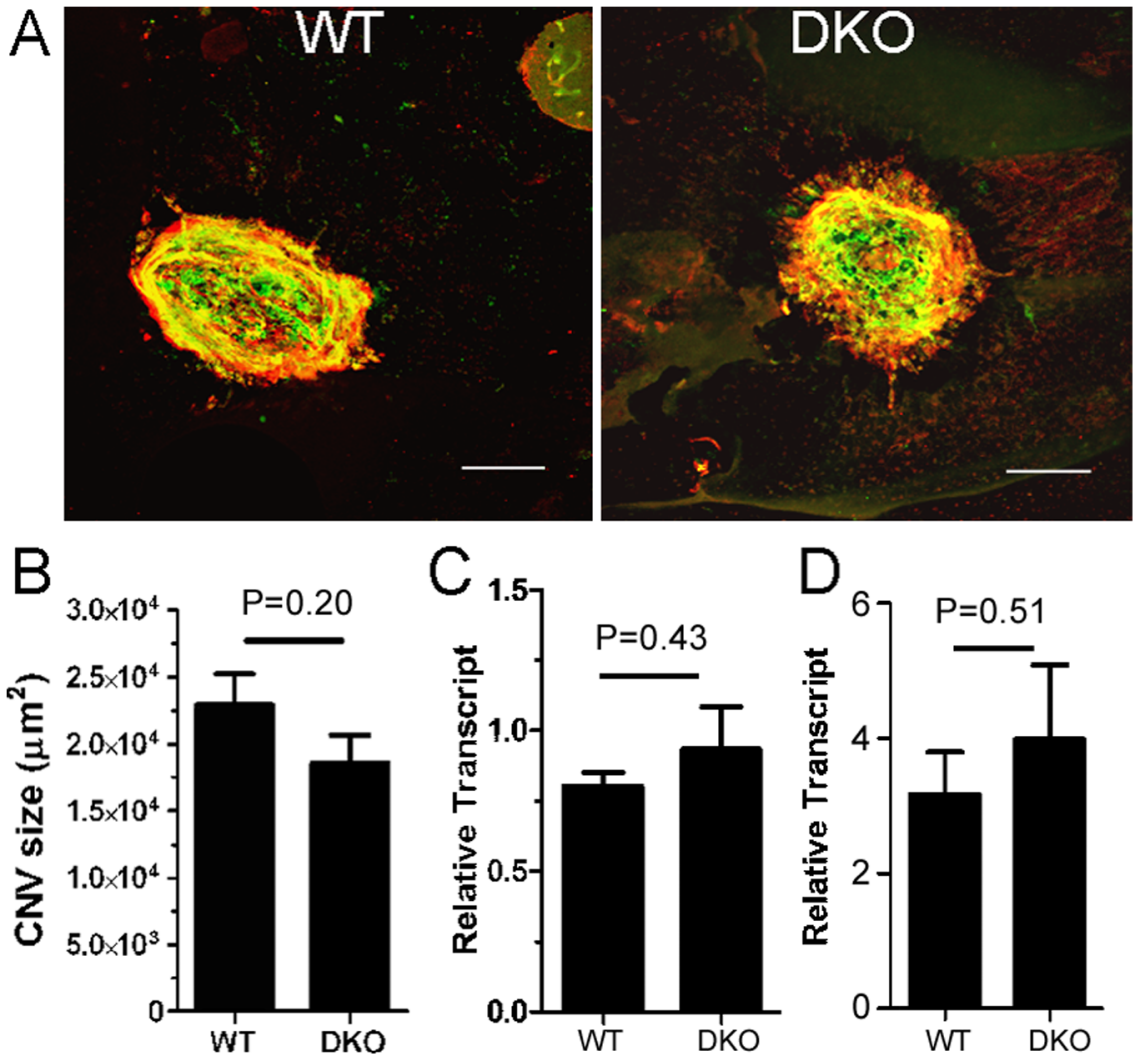
Choroidal neovascularisation in CCL2^−/−^CX3CR1^GFP/GFP^ DKO mice. (A) CNV was induced in adult (8∼12-week) WT and CCL2^−/−^CX3CR1^GFP/GFP^ mice. RPE/choroidal flatmounts were collected at day 10 and stained for isolectin B4 (green) and collagen IV (red) and examined by confocal microscopy. (B) CNV size in WT and CCL2^−/−^CX3CR1^GFP/GFP^ mice at day 10. N = 6. (C, D) VEGF-A mRNA expression in RPE cells (C) and BM-DMs (D) from WT and CCL2^−/−^CX3CR1^GFP/GFP^ mice. N = 5.

Indocyanine green angiography did not reveal any CNV in normal aged or 800lux light treated CCL2^−/−^CX3CR1^GFP/GFP^ mice (Figure 1G, 1H). When CNV was induced using 532 nm diode laser, the size of the CNV did not differ significantly between WT and CCL2^−/−^CX3CR1^GFP/GFP^ mice (Figures 2A, 2B). Furthermore, RPE cells (Figure 2C) and BM-DMs (Figure 2D) from WT and CCL2^−/−^CX3CR1^GFP/GFP^ mice express comparable levels of VEGF.

Our results suggest that the deletion of CCL2 and CX3CR1 predisposes mice to age- and light-mediated retinal damage, but does not affect pathogenic retinal angiogenesis.

### RPE Damage and Photoreceptor Degeneration in Aged and 800 Lux Chronic Light Exposed Mice

**Figure 3:**
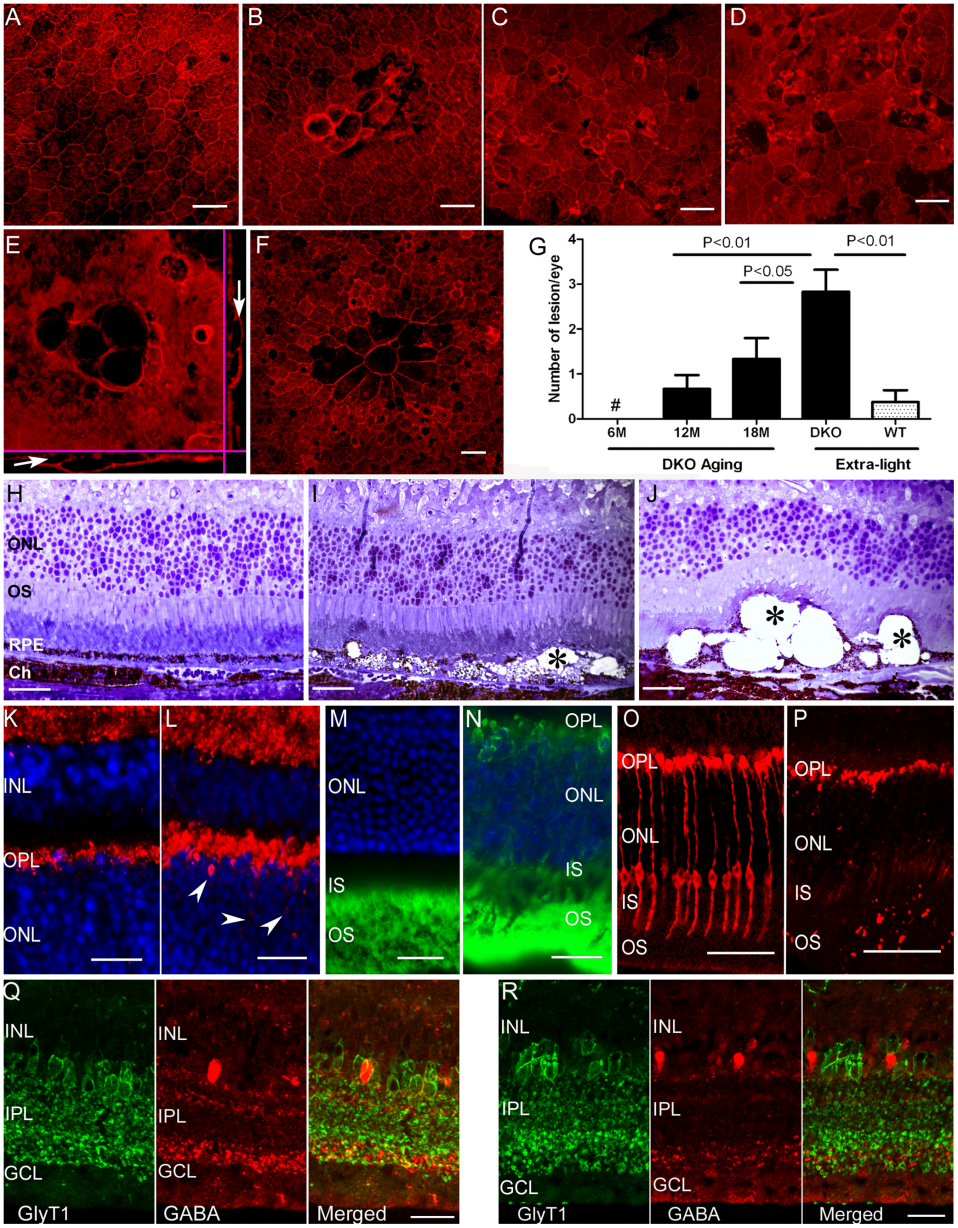
Retinal pigment epithelium (RPE) damage, photoreceptor degeneration and amacrine cell alteration in CCL2^−/−^CX3CR1^GFP/GFP^ mice. (A–F) Confocal images of RPE/choroidal flatmount from 12-month WT (A), 12-month CCL2^−/−^CX3CR1^GFP/GFP^ (B), 18-month CCL2^−/−^CX3CR1^GFP/GFP^ (C), and 10-month+light CCL2^−/−^CX3CR1^GFP/GFP^ (D, E) and 10-month+light WT (F) mice. Arrows in (E) indicate RPE vacuoles in z-stack images. Scale bar = 50 µm. (G) The average number of RPE lesion in different groups of mice. N ≥6, unpaired Student *t* test. #, no lesions were detected. (H–J) semithin retinal sections from 18-month WT (H), 18-month CCL2^−/−^CX3CR1^GFP/GFP^ (I) and 10-month+light CCL2^−/−^CX3CR1^GFP/GFP^ mice (J). ONL, outer nuclear layer; OS, outer segments; Ch, choroid. Asterisks indicate RPE vacuoles. (K, L) Synaptophysin expression in 18-month WT (K) and 18-month CCL2^−/−^CX3CR1^GFP/GFP^ (L) mouse retina. OPL, outer plexiform layer; INL, inner nuclear layer. Synaptophysin was observed in ONL in aged CCL2^−/−^CX3CR1^GFP/GFP^ mice (arrowheads, L). (M, N) Rhodopsin expression in 18-month WT (M) and CCL2^−/−^CX3CR1^GFP/GFP^ (N) mouse retina. Rhodopsin was detected in OPL and ONL layers in aged CCL2^−/−^CX3CR1^GFP/GFP^ mouse (N). (O, P) Cone arrestin expression in 18-month WT (O) and CCL2^−/−^CX3CR1^GFP/GFP^ (P) mouse retina. (Q, R) Glycine transporter 1 (green) and GABA (red) immunoreactivities in 12-month WT (Q) and CCL2^−/−^CX3CR1^GFP/GFP^ (R) mouse retina. A marked reduction in GABA expression was noted in the IPL of CCL2^−/−^CX3CR1^GFP/GFP^ mouse retina. GCL, ganglion cell layer. Scale bar = 25 µm.

The effects of age and 800 lux chronic light exposure on RPE morphology was assessed in flat mount preparations. F-actin was uniformly expressed at the cell-cell junctions in RPE cells in 6∼18-month old WT mice (Figure 3A), and in 6-month old CCL2^−/−^CX3CR1^GFP/GFP^ mice (data not shown). Patches of RPE abnormality evidenced by altered cell junction, loss of hexagonal tessellation and uneven distribution of F-action, were observed in 33% of 12-month old (Figure 3B) and 50% 18-month old (Figure 3C) CCL2^−/−^CX3CR1^GFP/GFP^ mice maintained under normal light-cycle conditions. The number of RPE lesion increased with age (Figure 3G). RPE lesions were observed in all light-treated CCL2^−/−^CX3CR1^GFP/GFP^ mice (n = 12 eyes, Figure 3D), however only 20% WT mouse eyes developed RPE lesion following 800 Lux light treatment (n = 20, Figure 3F). Multiple vacuoles with different sizes were observed in Z-stack confocal images in eyes from CCL2^−/−^CX3CR1^GFP/GFP^ mice (Figures 3C–3E). On average 2.83±0.48 lesions per eye were observed in 800 Lux light treated CCL2^−/−^CX3CR1^GFP/GFP^ mice and 1.33±0.46 lesions per eye were detected in 18-month old CCL2^−/−^CX3CR1^GFP/GFP^ mice housed under normal lighting conditions (Figure 3G). In light treated WT mice, approximately one lesion was detected in every three eyes (0.3±0.14 lesions per eye, Figure 3G).

Semithin sections revealed multiple vacuolisations and pigment loss in RPE cells from aged CCL2^−/−^CX3CR1^GFP/GFP^ mice (Figure 3I) but not in age-matched WT controls (Figure 3H). Large vacuoles, similar to that observed in RPE flatmount (Figure 3E) were observed in light-treated CCL2^−/−^CX3CR1^GFP/GFP^ mice (Figure 3J). Photoreceptor loss was observed in areas corresponding to severe RPE damage (Figure 3J). Synaptophysin, a synaptic vesicle glycoprotein, is expressed in the inner and outer plexiform layers of the retina (Figure 3K). In aged CCL2^−/−^CX3CR1^GFP/GFP^ mice, synaptophysin expression was extended to the outer nuclear layer (ONL, arrows in Figure 3L) of areas related to photoreceptor loss. Rhodopsin is located in photoreceptor outer segments (OS) and the expression was similar between WT and CCL2^−/−^CX3CR1^GFP/GFP^ mice at young ages (3–6 months, data not shown). In 18 months old mice, extensive rhodopsin expression was detected across the retina in the outer plexiform layer (OPL), ONL and photoreceptor inner segments (IS) of CCL2^−/−^CX3CR1^GFP/GFP^ (Figure 3N) but not WT mice (Figure 3M). Moreover, cone arrestin expression was markedly down-regulated throughout the retina of aged (18-month) CCL2^−/−^CX3CR1^GFP/GFP^ mice (Figure 3P) but not in WT mice (Figure 3O). GABA and GlyT1 immunohistochemistry was performed to analyse amacrine cells at the inner retina. GlyT1 immunoreactivity showed similar morphology and stratification of glycinergic amacrine cells in aged WT (Figure 3Q) and CCL2^−/−^CX3CR1^GFP/GFP^ mice (Figure 3R). However, a marked reduction in GABA expression was found across the IPL in DKO mice (compare Figures 3Q and 3R). The disrupted expressions of rhodopsin, cone arrestin, and GABA were observed across the entire retina of DKO mice, although the changes were more intense in the areas correlated to photoreceptor loss. Overall, these results suggest photoreceptor degeneration, synaptic structural remodelling and amacrine cell changes in aged CCL2^−/−^CX3CR1^GFP/GFP^ mice.

**Figure 4:**
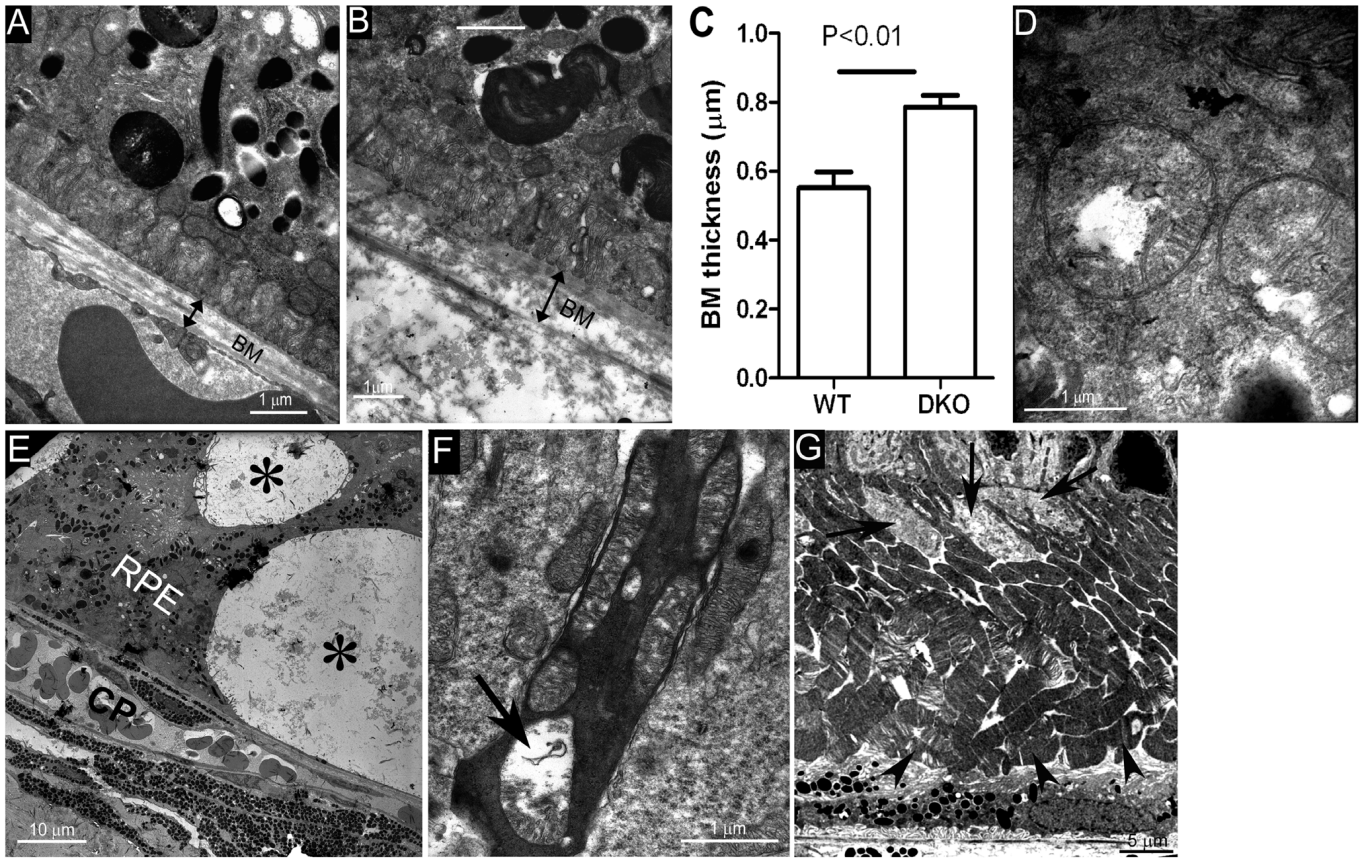
Transmission electron microscopic (TEM) images of mouse eyes. (A) Image from an 18-month WT mouse. (B) Image from an 18-month CCL2^−/−/^CX3CR1^GFP/GFP^ mouse. (C) The average thickness of Bruch membrane in WT and CCL2^−/−/^CX3CR1^GFP/GFP^ mice. N = 6 (eyes). (D) Vacuolated changes in RPE mitochondria from CCL2^−/−/^CX3CR1^GFP/GFP^ mice. (E) Vacuolated changes (asterisks) in RPE cells in CCL2^−/−^CX3CR1^GFP/GFP^ mice. (F) Photoreceptor inner segment damage (arrow) in CCL2^−/−^CX3CR1^GFP/GFP^ mice. (G) Photoreceptor inner segment damage (arrows) and outer segment disorientation (arrowheads) in an area without vacuolated RPE damage. BM, Bruch membrane; CP, choroidal capillaries; RPE, retinal pigment epithelium.

Electron transmission microscopy revealed an increased thickness of Bruch’s membrane in aged CCL2^−/−^CX3CR1^GFP/GFP^ mice (Figure 4B) compared to WT mice (Figure 4A, 4C). In addition, mitochondrial damage (Figure 4D), RPE vacuolization (Figure 4E) and photoreceptor damage (Figure 4F) were observed in aged and light-treated CCL2^−/−^CX3CR1^GFP/GFP^ mice. Although photoreceptor damage was more frequently observed in corresponding RPE vacuolated area, photoreceptor inner segment damage (arrows in Figure 4G) and outer segments disorientation (arrowheads, Figure 4G) were also observed in areas without RPE vacuolation.

### Retinal Immune Activation in CCL2^−/−^CX3CR1^GFP/GFP^ Mice

**Figure 5:**
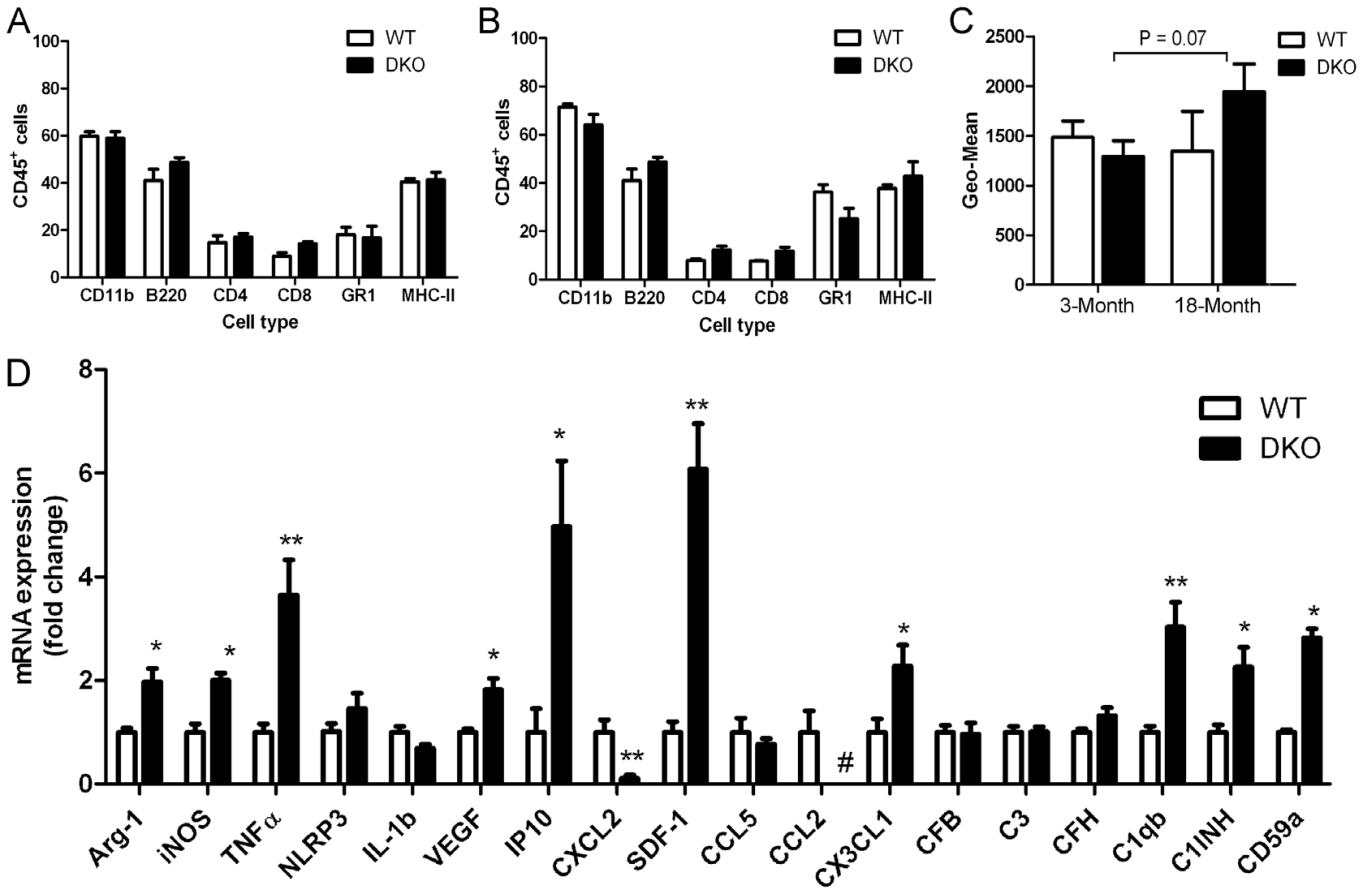
Immune response in WT and CCL2^−/−^CX3CR1^GFP/GFP^ mice. (A–C) flow cytometry analysis of blood cells (gated on CD45^+^ cells) from 3-month (A) and 18-month (B) old WT and CCL2^−/−^CX3CR1^GFP/GFP^ mice. (C) The expression levels of MHC-II (geo-mean) in CD45^+^ blood cells in different groups of mice. (D) Real-time RT-PCR analysis of retinal gene expression. *, P<0.05; **<0.01 compared to WT mice, n = 6, unpaired Student t test.

Flow cytometry analysis of blood samples showed that WT and CCL2^−/−^CX3CR1^GFP/GFP^ mice had comparable levels of various immune cells in both young (3-month, Figure 5A) and aged (18-month, Figure 5B) groups. The expression level of MHC-II in CD11b^+^ cells from aged CCL2^−/−^CX3CR1^GFP/GFP^ mice was slightly increased; however, the increment was not statistically significant (Figure 5C).

To understand whether the combined deletion of CCL2 and CX3XR1 affects retinal immune activation, genes involved in inflammation (iNOS, TNFα, NLRP3, IL-1β), complement activation (C3, CFB,CFH, C1qb, C1INH, CD59a) and wound healing or tissue remodelling (Arg-1, VEGFa) were selected for quantitative PCR analysis. In addition, chemokines CCL2, CCL5, CXCL2, CXCL10 (IP10), CXCL12 (SDF-1), and CX3CL1 that are known to be expressed by neuronal tissue and are involved in macrophage/microglial function were also selected. Increased mRNA expression was observed in a number of immune related genes in the retinas of CCL2^−/−^CX3CR1^GFP/GFP^ mice compared to those of WT mice (Figure 5D). The upregulated genes include *Arg-1, iNOS, NLRP3, TNFα, VEGF, CXCL10 (IP-10), CXCL12 (SDF-1), CX3CL1, C1qb, C1NIH*, and *CD59a* (Figure 5D). Interestingly, the expression of chemokine *CXCL2* was reduced in CCL2^−/−^CX3CR1^GFP/GFP^ mouse retinae (Figure 5D).

**Figure 6:**
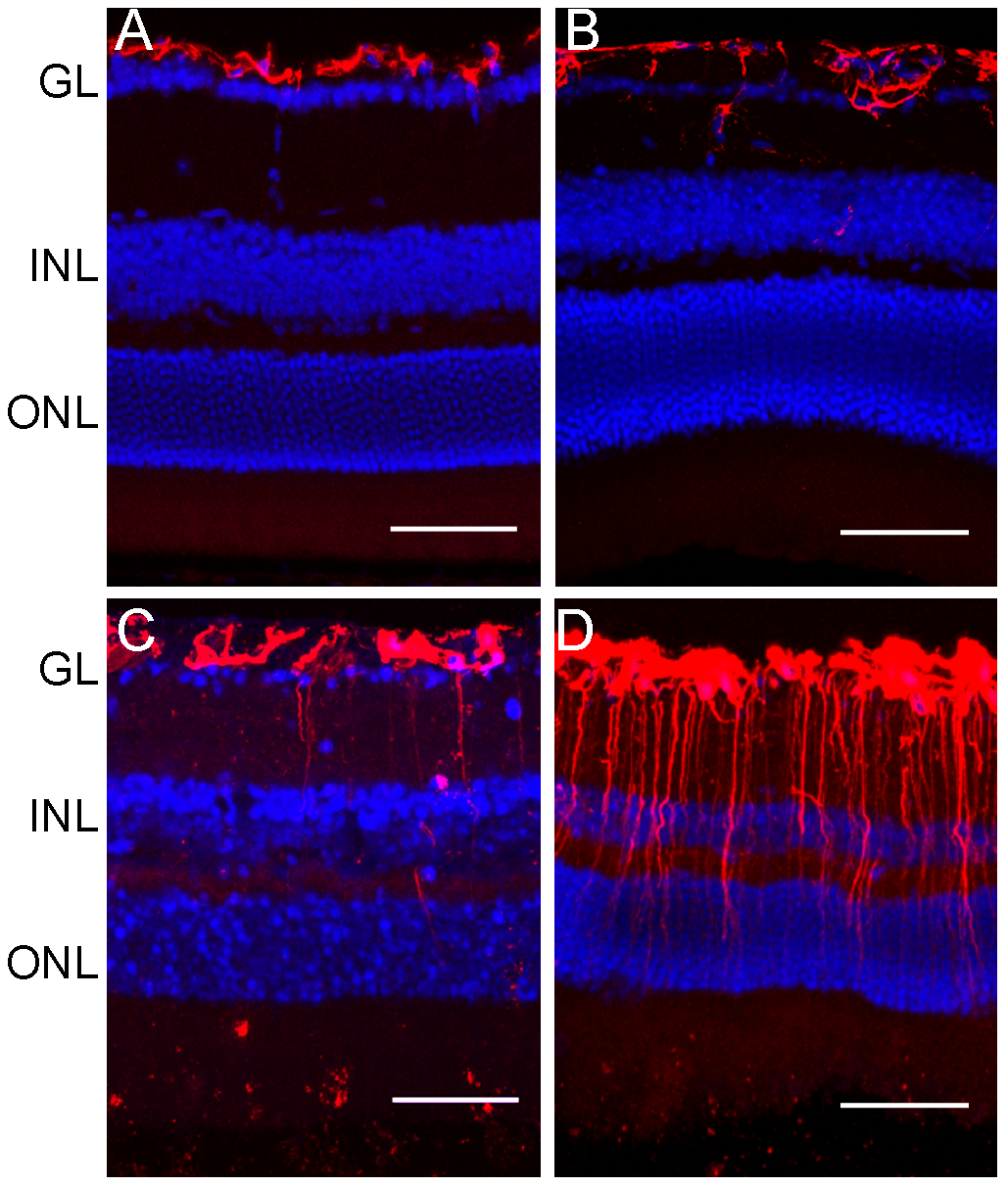
Retinal GFAP expression in WT and CCL2^−/−^CX3CR1^GFP/GFP^ mice. Cryosections of mouse eyes were stained for GFAP (red) and DAPI (blue) and observed by confocal microscopy. (A) 3-month WT mice. (B) 3-month CCL2^−/−^CX3CR1^GFP/GFP^ mice. (C) 18-month WT mice, (D) 18-month CCL2^−/−^CX3CR1^GFP/GFP^ mice. GL, ganglion layer; INL, inner nuclear layer; ONL, outer nuclear layer. Scale bar = 20 µm.

Immunofluorescent investigation of retinal sections showed that GFAP was expressed in the ganglion layer in 3-month old WT (Figure 6A) and CCL2^−/−^CX3CR1^GFP/GFP^ mice (Figure 6B), although expression in the latter slightly extended to the inner nuclear layer (INL, Figure 6B). In aged (18-month) WT (Figure 5C) and CCL2^−/−^CX3CR1^GFP/GFP^ mice (Figure 5D) the expression was significantly increased and extended to the outer nuclear layers. The increment was more significant in aged CCL2^−/−^CX3CR1^GFP/GFP^ mice (Figure 6D) compared to WT mice (Figure 6C).

**Figure 7:**
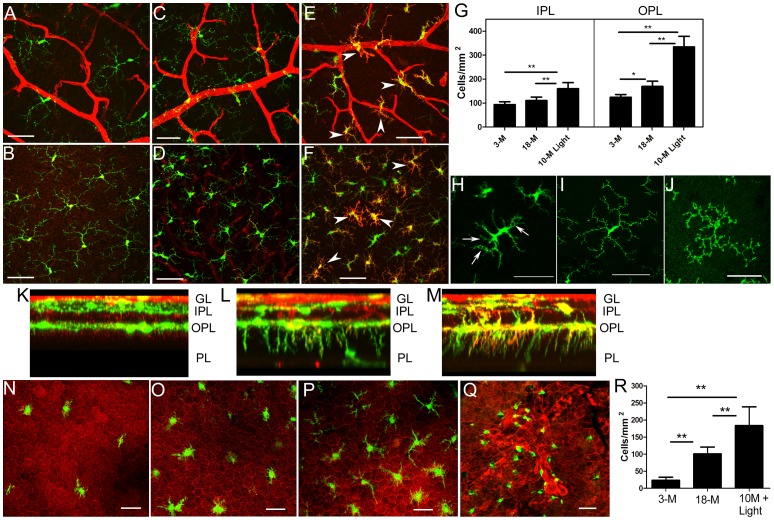
Retinal microglial activation in different ages of CCL2^−/−^CX3CR1^GFP/GFP^ mice. (A–F, K–M) Retinal flatmounts from DKO mice were stained for isolectin B4 (red) and examined by confocal microscopy. (A, C, E) Confocal images from inner plexiform layer (IPL). (B, D, F) Confocal images from outer plexiform layer (OPL). (A, B) 3-month old. (C, D), 18-month old. (E, F) 10-month+light-treatment. (G) The number of microglial cells in different groups of mice. (H) High magnification image showing activated microglia from light-treated mice. (I) High magnification image showing a naive resident microglia from a 3-month old DKO mouse. (J) High magnification image showing a CD11b^+^ naïve resident microglia from a 3-month old WT mouse. (K–M) Z-axils reconstruction of confoal z-stack images from a 3-month old (K), an 18-month old (L), and 10-month+light CCL2^−/−/^CX3CR1^GFP/GFP^ (M) mouse retina. (N–Q) RPE/choroidal flatmounts showing subretinal macrophages in a 3-month old (N), an 18-month old (O), and 10-month+light (P, Q) CCL2^−/−/^CX3CR1^GFP/GFP^ mouse eyes. RPE cells were stained with rhodamine-phalloidin (red). (R) The number of subretinal macrophages in different groups of mice. *, P<0.05; **<0.01, n = 6. GL, Ganglion layer; PL, photoreceptor layer. Scale bar = 50 µm.

In CCL2^−/−^CX3CR1^GFP/GFP^ mice, all microglial cells are GFP+. Confocal microscopy of retinal flatmounts showed that the number of microglial cells in both inner plexiform layer (IPL, Figures 7A, 7C,7E) and outer plexiform layer (OPL, Figure 7B, 7D, 7F) increased in 18-month old CCL2^−/−^CX3CR1^GFP/GFP^ mice, and chronic light-treatment resulted in significantly more microglial cells in the retina (Figure 7G). Furthermore, microglial cells in aged and chronic light-treated mice displayed shorter and larger dendrites and had bigger somas (Figure 7H) compared to microglia from 3-month old DKO mice (Figure 7I) and WT mice (Figure 7J). Microglial cells in chronic light-treated mice also expressed high levels of isolectin B4 (Figures 7E, 7F). Although all microglia cells in chronic light-treated mice displayed active morphology, the expression levels of isolectin B4 differed greatly in different cells (Figures 7E, 7F), suggesting heterogeneous phenotype of retinal microglia. In chronic light-treated mice, 15% of microglial cells contained one or more vacuoles (Figure 7H), possibly indicative of active phagocytosis. The aforementioned microglial activation was observed throughout the neuronal retina of aged and light-treated DKO mice.

When the whole thickness of the retina was scanned in z-stack images, GFP+ microglial cells were observed in ganglion layer (GL), IPL and OPL in 3-month old mice (Figure 7K). However, in 18-month old mice, many dendrites, and occasionally cell bodies were observed in the photoreceptor layer (PL, Figure 7L). In chronic light-treated mice, many more dendrites, some of which also express high levels of isolectin B4, were detected in the photoreceptor layer (Figure 7M).

In 3-month old CCL2^−/−^CX3CR1^GFP/GFP^ mice, a few GFP+ microglial cells were detected in RPE/choroidal flatmounts (Figure 7N), and the number of subretinal microglia increased significantly in 18-month CCL2^−/−^CX3CR1^GFP/GFP^ mice (Figures 7O, 7R). Many more subretinal macrophages were detected in the eyes from chronic light-treated CCL2^−/−^CX3CR1^GFP/GFP^ mice (Figures 7 P–R). At the RPE lesion sites macrophages displayed small cell bodies and less or no dendrites (Figure 7Q).

The results suggest that deletion of CCL2 and CX3CR1 results in a greater retinal immune activation under chronic stress conditions.

### Altered Immune Activation in Bone Marrow–Derived Macrophages of CCL2^−/−^CX3CR1^GFP/GFP^ Mice

**Figure 8:**
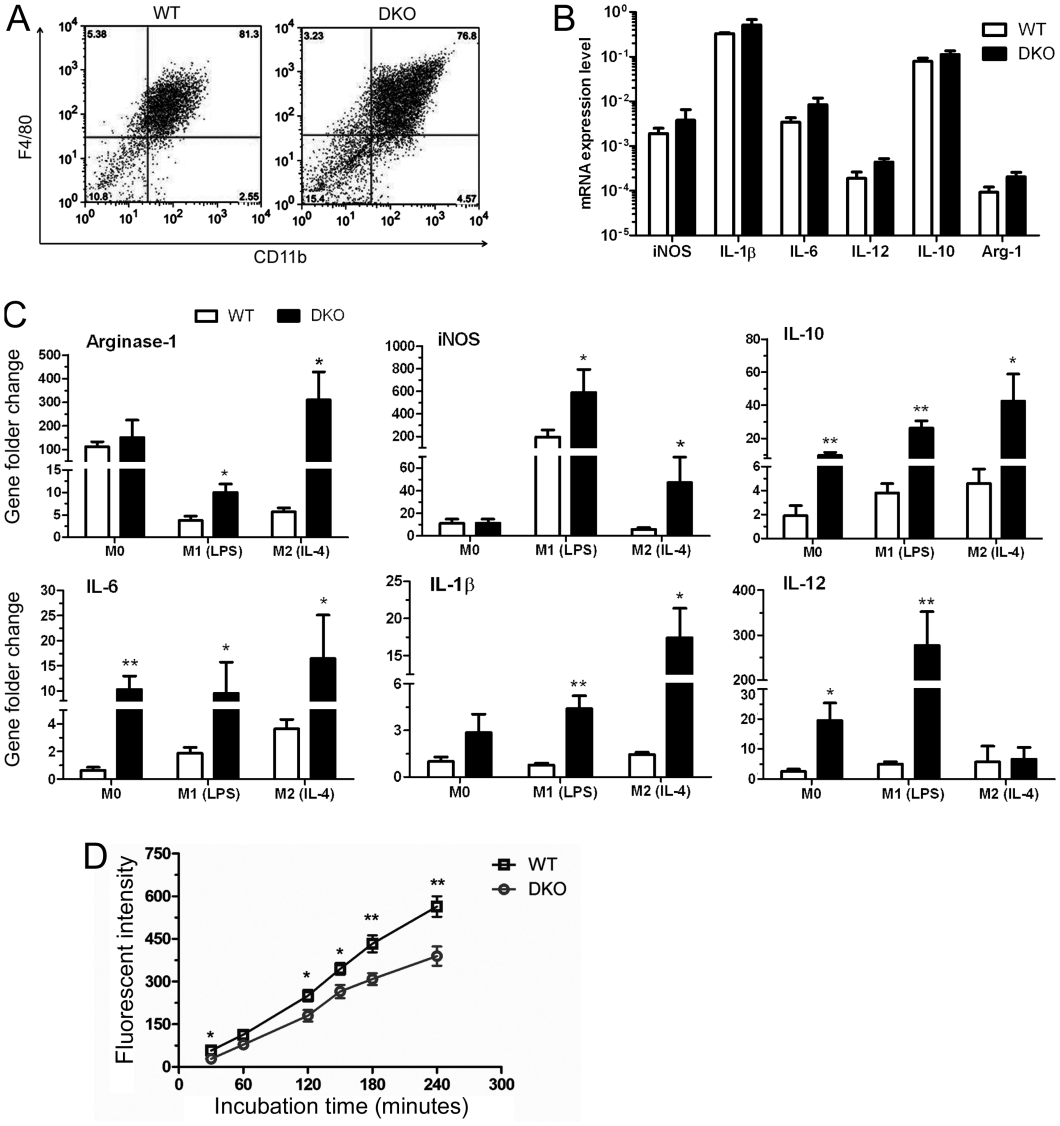
Macrophage phenotype and function in CCL2^−/−^CX3CR1^GFP/GFP^ DKO mice. Bone marrow-derived macrophages (BM-DMs) were cultured from WT and CCL2^−/−^CX3CR1^GFP/GFP^ mice (see Materials & Methods). Cells were then used for gene expression and phagocytosis assay. In some experiments, BM-DMs were further polarized into M1 (with LPS) or M2 (with IL-4) macrophages. (A) Flow cytometry analysis showing F4/80 and CD11b expression in BM-DMs. (B) Real-time RT-PCR analysis of immune related genes in undifferentiated M0 macrophages from WT and DKO mice under normoxia culture condition. Gene expression level was normalised by housekeeping gene (18s). (C) Real-time RT-PCR analysis of immune related genes in different subsets of BM-DMs (M0, M1, and M2) from WT and DKO mice under hypoxia conditions (1% oxygen). Data shown are mRNA fold changes versus WT M0 BM-DMs under normoxia conditions. *, P<0.05; **<0.01, compared WT BM-DMs, n = 3. (D) Phagocytosis of M0 BM-DMs from WT and DKO mice determined by the pHrodo™ E. coli Bioparticles® Phagocytosis assay. *, P<0.05; **<0.01, compared to DKO BM-DMs at the same time point. N = 6.

To further understand the underlying mechanism related to enhanced retinal immune activation in CCL2^−/−^CX3CR1^GFP/GFP^ mice, we investigated the immune function of myeloid-derived cells. The phenotype of bone marrow-derived macrophages (BM-DMs) from WT and CCL2^−/−^CX3CR1^GFP/GFP^ mice did not differ (Figure 8A). They also expressed similar levels of various immune mediators, including *iNOS, Arginase-1, IL-6, IL-1β, IL-12 (p40)* and *IL-10* (Figure 8B). The retina has the lowest levels of oxygen tension in the body (PO_2_∶5–25 mmHg in the retina compared to 85–90 mmHg in systemic arterial circulation) and is considered to have a hypoxic microenvironment [45]. To mimic the in vivo situation, we cultured BM-DMs under hypoxic conditions (1% O_2_). Interestingly, BM-DMs from CCL2^−/−^CX3CR1^GFP/GFP^ mice expressed significantly higher levels of *iNOS, IL-6, IL-1β, IL-12,* and *IL-10*, particularly when they were polarized to a classically activated inflammatory macrophage phenotype with LPS (M1) or alternatively activated wound-healing macrophage with IL-4 (M2) (Figure 8C). In addition, BM-DMs from CCL2^−/−^CX3CR1^GFP/GFP^ mice also had significantly reduced phagocytic activity compared to those from WT mice (Figure 8D).

The results suggest that the deletion of CCL2 and CX3CR1 results in greater immune activation but lower scavenging activity in myeloid-derived cells under stress conditions.

## Discussion

In 2007, Tuo et al reported that the combined deletion of chemokine *Ccl2* and Fractalkine receptor *Cx3cr1* on C57BL/6N genetic background (i.e., CCL2^−/−^CX3CR1^−/−^ mice) resulted in an early onset of spontaneous retinal degeneration with AMD-like features such as RPE alteration, photoreceptor damage and A2E elevation [30], and the mouse has been considered as a valuable model of AMD [46]. A recent study has shown that the C57BL/6N mice have *Crb-1 rd8* mutation [31], and it has been suggested that retinal lesions in the Ccl2^−/−^Cx3cr1^−/−^ mice may be caused by *rd8* mutation rather than *Ccl2* and *Cx3cr1* deletion [31], [32]. Further, Vessey et al observed inner retinal dysfunction related to amacrine abnormality but no retinal degeneration in adult (up to 9 months) DKO mice [35]. More recently, Luhmann et al reported that when mice were housed under dim conditions (33 lux light intensity), none of the genetically modified mice, including CCL2 KO, CX3CR1 KO and CCL2/CX3CR1 DKO developed retinal degeneration by the age of 12–14 months [34]. In this study, we show that CCL2^−/−^CX3CR1^GFP/GFP^ mice on C57BL/6J genetic background age-dependently develop localised retinal atrophy, akin to human GA, and the minimal age of developing GA-like lesions is 12 months. Chronic light exposure further enhances the size and number of retinal lesions (Figure 3G). Since our mice do not have *Crb-1 rd8* mutation, our results suggest that the deletion of *Ccl2* and *Cx3cr1* predisposes mice to retinal atrophy under aging and chronic light damage conditions more so than in the single CCL2 or CCR2 knockout mice [28], [29]. Inner retinal (amacrine cell) abnormalities were also observed in our study. An explanation for the discrepancies in our data on outer retinal degeneration in the DKO mice and that of others remains elusive. It is possible that different animal housing conditions and different techniques used to detect lesions may be responsible for the different results. CCL2-CCR2 and CX3CL1-CX3CR1 are important chemokine pathways involved in various immune responses. Immune responses only occur when tissues suffer from exogenous pathogen invasions or endogenous “danger” insults. Luhmann et al housed their mice under dim conditions (33 Lux light intensity) [34]. The low levels of light exposure may not cause sufficient oxidative damage to the eye even up to 12–14 months of age. In the absence of retinal insults, inflammation will not occur and the effects of CCL2 or CX3CR1 deletion will not be detectable. Furthermore, we detected 1.33 lesions/eye (with an average size equivalent to that of the optic disc) in 18 month old mice housed under normal lighting conditions by confocal microscopy of RPE flatmounts (Figure 3G). The low numbers of small lesion can easily be missed in retinal histological investigations (the techniques that other studies applied).

Oxidative damage is considered one of the triggers for age-related retinal degeneration. A healthy immune system helps to remove damaged molecules and dead cells and to promote tissue remodelling by mounting a para-inflammatory response [47]. Under normal aging conditions, if the level of age-related retinal damage and the homeostatic capacity of para-inflammation are balanced, the risk of retinal pathology may be avoided. We have shown previously that age-related retinal para-inflammatory responses are expressed by resident microglia and RPE cells [24] as well as bone marrow-derived myeloid cells [27]. Increased oxidative damage resulting from environmental risk factors may break the balance leading to lesion development. By exposing mice to 800 Lux additional lights, the levels of oxidative stress to the retina were increased. In WT mice, where the para-inflammatory response is tightly controlled, the majority of mice were able to maintain retinal homeostasis (evidenced by the low incidence of disease and low number of lesions (0.30±0.14). All light-treated CCL2^−/−^CX3CR1^GFP/GFP^ mice developed GA-like lesions and more lesions occurred in each eye (2.83±0.48). It appears that the cumulative effect of age, light damage and *Ccl2/Cx3cr1* gene deletions combine to break the balance and increase the likelihood of GA-like lesions.

In addition to oxidative insults, dysfunction in any of the cells involved in retina para-inflammation, or dysregulation of any of the immune responses may also disturb the balance resulting in GA-like lesions in the aging retina. Under normal housing conditions, the systemic immunological parameters between WT and CCL2^−/−^CX3CR1^GFP/GFP^ mice did not differ, whereas retinal immune response was significantly increased in CCL2^−/−^CX3CR1^GFP/GFP^ mice as compared to that in WT mice. The CX3CL1-CX3CR1 pathway is known to play an important role in regulating microglial activation [48], and is neuroprotective [48], [49]. More recently, we have shown that in the absence of CX3CR1, ischemia and oxidative damage-mediated retinal degeneration is accelerated [50]. The lack of CX3CR1 may be responsible for the increased microglial activation and retinal degeneration in the DKO mice under ageing and light exposure conditions. In addition, we detected increased levels of CXCL10 (IP-10) and CXCL12 (SDF-1) in DKO mouse retina (Figure 5). Although the underlying mechanism related to over expression of these CXC chemokines remains elusive, they may be involved in excessive retinal microglial activation. The contribution of CCL2 depletion to age- and light-mediated retinal degeneration may be related to altered monocyte and macrophage function in these mice. Previously, we have shown that macrophages from CCL2 KO mice express excessive amounts of inflammatory mediators such as inducible nitric oxide synthase (iNOS) and TNF-α [28]. In this study, TNF-α (along with chemokines CXCL10 and CXCL12) mRNA expression was significantly increased in DKO mouse retina (Figure 5). Although under normal culture conditions, BM-DMs from WT and CCL2^−/−^CX3CR1^GFP/GFP^ mice had a similar phenotype and expressed comparable levels of inflammatory cytokines/chemokines, when these cells were cultured under hypoxic conditions (1% O_2_), cells from CCL2^−/−^CX3CR1^GFP/GFP^ mice expressed significantly higher levels of inflammatory mediators (Figure 8). The retina has the highest metabolic activity within the body, and is constantly exposed to relatively hypoxic conditions [45]. The retina may also suffer from higher levels of oxidative stress compared to other tissues due to sustained light exposure. Our results suggest that the combined deletion of *Ccl2* and *Cx3cr1* results in aggressive inflammatory responses by monocytes and macrophages under hypoxia and stress-related conditions.

Bone marrow-derived macrophages from CCL2^−/−^CX3CR1^GFP/GFP^ mice have significantly reduced phagocytic activity compared to those from WT mice. Previously, we have observed similar phenomena in CCL2 KO and CCR2 KO mice [28]. CCL2-CCR2 pathway may contribute to altered myeloid cell function in CCL2^−/−^CX3CR1^GFP/GFP^ mice. The physiological role of para-inflammation is to remove damaged molecules and dead cells to maintain tissue homeostasis [24], [47], and phagocytosis is essential to achieve this goal. Although monocytes/microglial cells in CCL2^−/−^CX3CR1^GFP/GFP^ mice are more active than cells in WT mice, they are less capable of removing damaged molecules and dead cells. Instead, they may produce higher levels of inflammatory mediators such as nitric oxide, IL-1β, and TNF-α, which may further damage tissue cells contributing to lesion development. Thus a possible explanation for the increased damage is one of dysregulated para-inflammatory responses in the DKO mice since they are less able to deal with the challenges of light exposure and age as well as WT mice.

RPE cell damage presents as localised “patchy” retinal lesions in this model. A recent study by Tarallo et al has shown that *Alu* RNAs (related to the loss of DICER1) induced NLRP3 inflammasome activation and its related IL-18 production play a key role in RPE cell death in GA [51]. In addition to *Alu* RNA, oxidised lipid can also activate NLRP3 inflammasome in RPE cells [52]. In this study, we detected an increased NLRP3 mRNA expression in aged WT and DKO mouse RPE compared to young mouse retina (data not shown). However, there is no significant difference between WT and DKO mouse retinas. Further studies are needed to understand whether excessive inflammasome activation exists in DKO RPE cells, and such studies are on-going in the investigators’ laboratory.

In summary, in this study, we show that disruption in the balance between the level of age-related retinal oxidative insults and the homeostatic capacity of the retinal para-inflammatory response to deal with them results in localised retinal atrophic lesions. More importantly, our data suggest that altered immune functions in the forms of uncontrolled microglial activation, reduced phagocytosis and increased inflammatory gene expression in immune cells may contribute to the development of a GA-like lesion during aging. Although AMD patients do not have CCL2 and CX3CR1 deficiency, many of them do have other immune-related gene polymorphisms, including genes involved in the complement system [53], TLRs [15], [54], and CX3CR1 [16], [17], [19]. Alterations in these genes may affect physiological “housekeeping” functions of innate immune cells such as phagocytosis of apoptotic dead cell debris and inflammatory gene expression, which may result in dysfunction or dysregulation of retinal para-inflammatory mechanisms [47], and ultimately AMD lesion development. While the current experiments go some way towards supporting this concept, we recognise that there are a number of limitations to the model. Firstly, the GA-like lesion mirrors only part of dry AMD pathology (e.g. RPE cell death and photoreceptor degeneration), and we did not observe drusen. Secondly, under normal ageing conditions only 50% of the mice develop the disease and the number of lesions in each eye is low. Therefore, specialised techniques such as RPE flatmount confocal microscopy are required to detect the lesion. However, the fact that the incidence and severity of disease can be manipulated by altering environmental risk factors known to be associated with AMD (e.g. light) suggests that comparable mechanisms are at play in this model as in human AMD. Therefore, we believe that despite the limitations, the CCL2^−/−^CX3CR1^GFP/GFP^ mouse is a useful model to dissect the immunopathogenesis of and to test therapeutic agents in dry AMD.
